# Oxygen-Dependent Light-Induced Lipid Membrane Perturbation by TLD1433 as a Potential Contributor to Phototoxicity

**DOI:** 10.1021/acs.inorgchem.6c01825

**Published:** 2026-07-20

**Authors:** Alessandra G. Ritacca, Abdelazim M. A. Abdelgawwad, Alisher Talgatov, Colin G. Cameron, Sherri A. McFarland, Antonio Francés-Monerris, Marta E. Alberto

**Affiliations:** † Dipartimento di Chimica e Tecnologie Chimiche, 18950 Università della Calabria, Arcavacata di Rende (CS) 87036, Italy; ‡ Institut de Ciència Molecular, 16781 Universitat de València, P.O. Box 22085, València 46071, Spain; § Department of Chemistry and Biochemistry, 12329 The University of Texas at Arlington, Arlington, Texas 76019-0065, United States

## Abstract

TLD1433 (Ruvidar®) is a Ru­(II) polypyridyl complex currently in phase II clinical trials for the treatment of non-muscle invasive bladder cancer using photodynamic therapy (PDT). Previous studies have demonstrated that TLD1433 exhibits dual Type I/II photosensitization, arising from a combination of a prolonged intrinsic excited state lifetime and the redox-active α-terthienyl group, which together enable both singlet oxygen sensitization and light-driven redox processes. Despite this, molecular determinants contributing to its pronounced phototoxicity are still being elucidated. Herein, we investigated whether light-induced lipid membrane perturbation could represent a contributing photochemical property relevant to cytotoxic activity. To this end, all-atom molecular dynamics simulations and NMR experiments were employed to examine the insertion, partitioning, and residence of TLD1433 within a lipid bilayer in both dark and photoactivated states in normoxia and hypoxia, as well as the resulting effects on membrane structure integrity. Under normoxic conditions, oxidation events from the triplet excited state of TLD1433 promote localized accumulation of oxidized lipids and induce membrane deformations characterized by increased water penetration and transient pore-like features. In contrast, under low ROS-mediated lipid oxidation, membrane perturbations are more limited, although increased permeability and structural disorder are still observed. Taken together, these results suggest that light-induced lipid membrane perturbation, including poration, may represent a contributing factor to the phototoxic effects of TLD1433 under normoxic conditions. This combined computational and experimental study details how the photochemical properties of TLD1433 influence membrane response in a light- and oxygen-dependent manner, offering a framework for understanding how such effects may contribute to PDT efficacy at an unprecedented molecular level.

## Graphical abstract

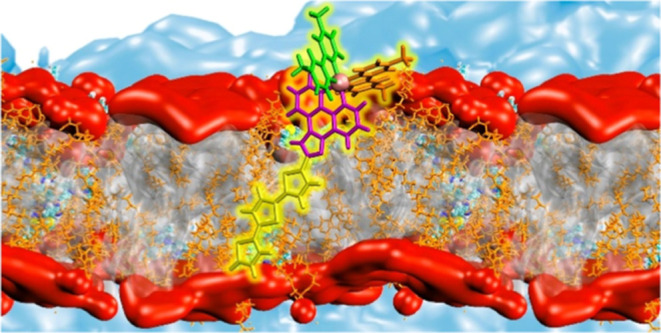

## Introduction

In the ongoing battle against cancer, photodynamic therapy (PDT) represents one of the most promising therapeutic strategies, thanks to its unique combination of low invasiveness, reduced toxicity and high spatiotemporal control over cytotoxicity that enhances tumor selectivity.^1–8^ PDT is among the most advanced light-driven approaches against cancer and requires a photosensitizer (PS), oxygen (O_2_) and a proper source of light within the therapeutic window (500–800 nm, approximately), to initiate the key photodynamic process responsible for the *in situ* reactive oxygen species (ROS) generation. The resulting local photocytotoxicity and tumor destruction is also accompanied by PDT’s well-documented ability to induce immune-mediated eradication of metastases and long-lasting prevention of recurrence.^9–15^

Although inherently complex, the main photodynamic effect that results in the damage or destruction of living tissues ultimately triggering apoptosis and/or necrosis, mainly stems from two quenching mechanisms of the PS excited triplet state (^3^PS): an energy transfer reaction from the excited ^3^PS to ground-state ^3^O_2_ to produce highly oxidant and cytotoxic singlet oxygen (^1^O_2_, Type II), or an electron–transfer reaction to yield superoxide (O_2_^•–^) and other ROS in a downstream cascade (H_2_O_2_ and OH^•^, Type I).^16^ It is well recognized that Type II PDT relies entirely on physiological oxygen levels, a condition frequently missing in the most aggressive and drug-resistant solid tumors. Moreover, oxygen availability can be further depleted by the vascular collapse induced by PDT itself, thereby exacerbating hypoxia conditions.^17^

For the Type I mechanism, oxygen-dependence is notably reduced^18–20^ since the generated ROS can participate in superoxide dismutase reactions that partially recycle O_2_, thereby sustaining ROS production even at low oxygen tensions.^21–23^ This has driven increasing interest in the design of Type I/Type II photosensitizers capable of switching between mechanisms depending on local oxygen concentration.^24–27^

Beyond these two well-established pathways, oxygen-independent processes, referred to in some literature as “Type III” mechanisms, have also been proposed. In these processes, the excited triplet state of the photosensitizer (^3^PS) interacts directly with biologically active molecules such as membranes, proteins, nucleic acids or lipids through contact-dependent photochemical events, leading to functional disruption.^16^ Importantly, these processes are more appropriately viewed as oxygen-independent extensions of Type I photochemistry rather than as a distinct mechanistic class and require effective localization of the PS at the biological target. As such, they remain operative under both normoxic and oxygen-depleted conditions.

Light-responsive metal complexes play a crucial role in this field, as the appropriate combination of metal centers and ligands enables access to a range of excited-state configurations with distinct photophysical characteristics.^28–30^ Moreover, rational modulation of the molecular scaffold allows for precise tuning of the photochemical, photophysical and biological properties of photoactive drugs. A wide variety of metal-based agents have therefore been proposed to address tumor hypoxia. One common strategy involves the photorelease of bulky ligands, generally based on Pt­(II) or Ru­(II) complexes acting as photoactivated chemotherapeutics (PACT).^29,31–33^ This intrinsic oxygen-independent approach is reminiscent of the mechanism of cisplatin and its derivatives, while offering light-mediated spatial and temporal control. Other strategies include photocaging of chemotherapeutics and enzyme inhibitors,^34,35^ as well as PSs with combined PDT/PACT activity.^36–100^

Among the investigated metals, the interest toward Ru­(II) complexes has increased markedly in recent years, particularly following the advancement of the terthienyl-containing Ru­(II) polypyridyl complex TLD1433 (Ruvidar®) into phase II clinical trials for treating non-muscle invasive bladder cancer (NMIBC) using PDT^28,43^ TLD1433 exhibits dual Type I/II photoreactivity, which has been linked to its pronounced phototoxicity across different oxygenation levels.^28,44^ Previous studies on this complex, and on several related analogue families,^28,44–49^ have identified the nondissociative triplet intraligand charge transfer (^3^ILCT) state as a key excited state responsible for its exceptional photoactivity, including under hypoxic conditions for some derivatives.^49–51^ The intrinsically long lifetime of the ^3^ILCT state, together with the redox-active terthienyl moiety, provides a mechanistic basis for the observed dual Type I/II behavior. However, how well these defined photophysical properties translate into specific biological damage pathways following green-light irradiation remains an active area of investigation and motivates further study.

Herein, the interaction of TLD1433 with lipid membranes at the molecular scale is investigated using atomistic molecular dynamics (MD) simulations. The primary objective is to examine the insertion, partitioning, and residence of TLD1433 within a lipid bilayer in both dark and light, and to evaluate how these interactions influence membrane structural properties. Different levels of ROS-mediated lipid oxidation were considered to mimic normoxic and hypoxic conditions and to reproduce the experimentally observed dependence of lipid oxidation on oxygen by TLD1433. Taken together, these results identify and characterize a membrane-based photoresponse, providing molecular-level insight into how the photophysical properties of TLD1433 may contribute to its phototoxic activity, as well as in related compounds,^47,49^ and informing the rational design of future phototherapy agents.

## Computational Details

### Model Systems Description

The all-atom models considered in this work, along with composition, number of replicas and total simulation times, are summarized in Figure 1 and Table S1. Cell membrane models were composed of 1-palmitoyl-2-oleoyl-phosphatidylcholine (POPC), whereas different extents of ROS-mediated lipid oxidation were modeled by randomly replacing POPC residues with 1-palmitoyl-2-(9′-oxo-nonanoyl)-*sn*-glycero-3-phosphocholine (POxPC) units (Figure S1),^48,49^ a major oxidation product of POPC, selected as a representative and well-characterized oxidized phospholipid for the membrane simulations.^52,53^ Indeed, the oxidation caused by ROS occurs on the single double bond present on C9 carbon of the acyl chain (Figure S1), whose mechanism involves the cleavage of such unsaturation and the introduction of the terminal polar group. POPC bilayers are widely used as a model of eukaryotic membranes in the liquid-disordered phase due to their zwitterionic nature and well-characterized structural and dynamical properties. This widespread use makes it a convenient and reliable reference system for investigating drug–membrane interactions.^52,54–57^

**Figure 1:**
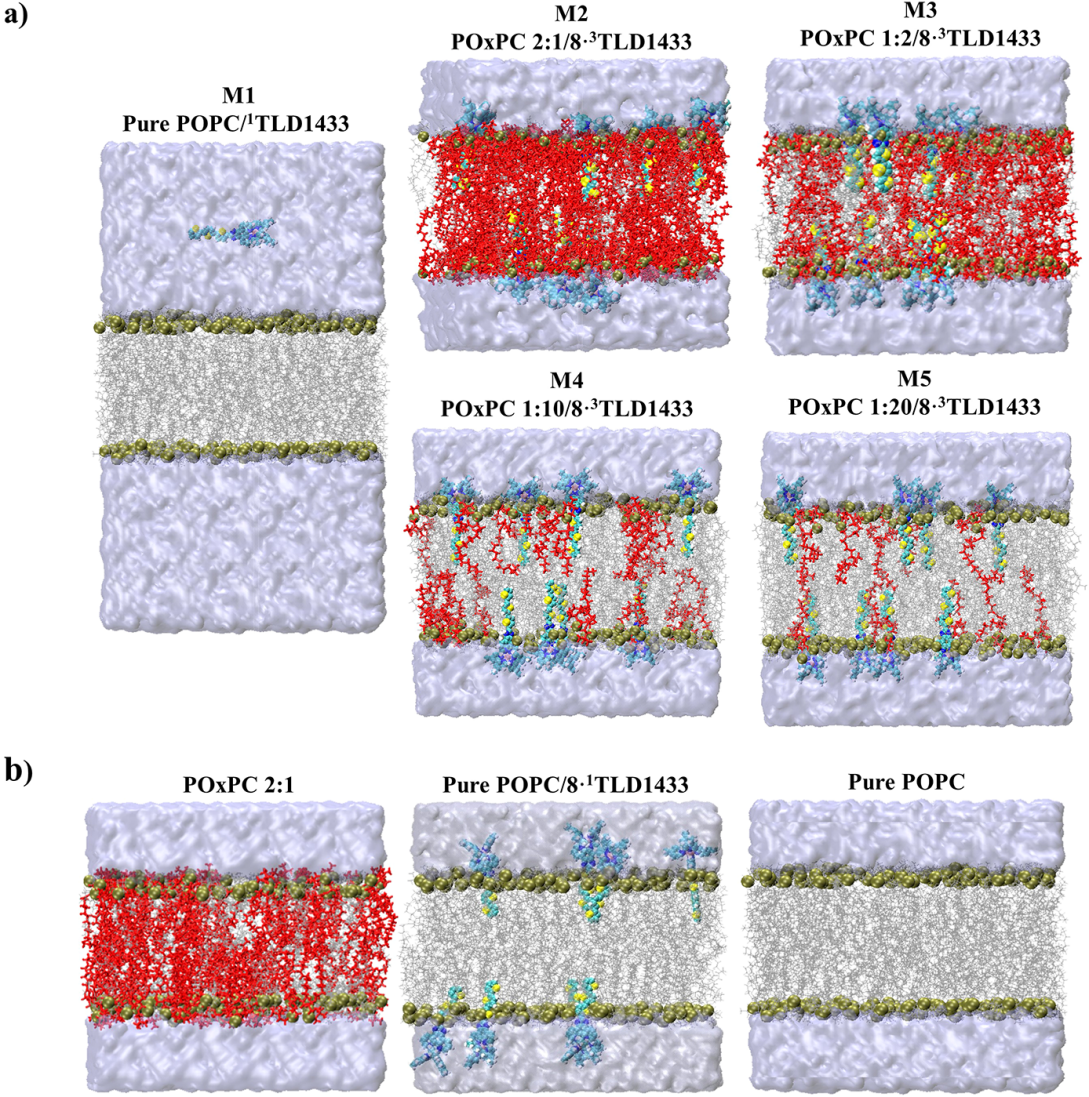
Starting coordinates of the pre-equilibrated lipid structures under investigation and with different ratios of POxPC/POPC lipids. (a) Problem systems: **M1**: One TLD1433 molecule in a pure POPC membrane at its ground state geometry, immersed in the bulk of water, to explore the insertion mechanism; **M2-M5**: Oxidized membrane models with different POxPC/POPC ratios, with eight embedded ^3^TLD1433 molecules at their T_1_ states; **M2**:POxPC/POPC 2:1; **M3,4,5**: POxPC/POPC 1:2, 1:10 and 1:20, respectively; (b) Control systems: POxPC 2:1 denotes a membrane with a POxPC/POPC ratio of 2:1; Pure POPC/8 ^1^TLD1433 denotes a pure POPC membrane with eight TLD1433 in their singlet S_0_ state; Pure POPC indicates a non-oxidized membrane model. TLD1433 molecules are represented as spheres, P atoms as tan spheres, POxPC as red licorice and POPC lipids as silver lines.

Systems containing TLD1433 molecules, either in S_0_ or T_1_, are considered as study systems (models **M1-M5**, see Figure 1 and Table S1). **M1** includes one TLD1433 molecule in S_0_ in a pure POPC membrane to simulate the insertion process in the dark. **M2-M5** are representative models to study the effect of the drug upon irradiation considering increasing hypoxia levels, represented by decreasing quantities of oxidized lipids in the membrane. In particular, eight TLD1433 molecules in their ^3^ILCT (T_1_) state were positioned symmetrically (four per leaflet) within the membrane models. The POxPC/POPC ratios in models **M2**-**M5** were 2:1, 1:2, 1:10, and 1:20, respectively.

Standalone membranes, either canonical (pure POPC) or oxidized (POxPC 2:1) were used as control systems, together with a model of a pure POPC membrane with eight TLD1433 molecules in the S_0_ ground state embedded to simulate dark conditions (Figure 1b).

### Membrane Models

Membrane models were built placing the lipid molecules, equally distributed between the two leaflets, in the middle of rectangular prisms of dimensions 80·80·80 Å, except for **M1** system, for which box dimensions are 80·80·140 Å, and filled with water and physiological concentrations of counterions ([KCl] = 0.15 M in all cases) to neutralize the total electric charge of the system. In the case of POPC membranes, starting coordinates were generated with the CHARMM-GUI web server^58^ using pre-equilibrated lipid structures,^59^ whereas oxidized membranes were generated with the recently updated CHARMM-GUI library.^60^ Membrane structural properties have been evaluated by using the MEMBPLUGIN integrated in the VMD visualization software.^61^.

### TLD1433 Description and MD Simulations Parameters

TLD1433 force–field parameters were obtained through the recently developed easyPARM tool.^62,63^ Metal–ligand parameters were derived from the vibrational frequencies computed with the widely used B3LYP functional as implemented in Gaussian 16 ^64^ with the 6-31G* basis set for all atoms, except for ruthenium (Ru), which was described using the quasi-relativistic Stuttgart–Dresden pseudopotential (SDD). Parameters of the organic ligands were retrieved from GAFF2 ^65^ and subsequently converted to the CHARMM General Force Field (CGenFF)^66^ format through the easyPARM built-in conversion tool.^62^

The restricted and unrestricted DFT formalisms were used to describe the closed- and open-shell S_0_ and T_1_ states of TLD1433, respectively. The same level of theory was used to compute atomic charges using the restricted electrostatic potential (RESP) method.^67^ The only differences between the S_0_ and T_1_ parameters of TLD1433, which in turn can influence the dynamics, are the atomic charges, the equilibrium bond distances and angles, and the corresponding force constants. Table S2 lists the atomic RESP charges computed for both electronic states, including the difference between them. For the vast majority of atoms, the charge difference caused by the ILCT excitation is smaller than 0.05, indicating that, overall, the impact of the triplet state in the TLD1433 parametrization is limited.

All simulations were run with the NAMD v3.0 code^68^ using the CHARMM force field for lipids^69^ and the TIP3P water model.^70^ Covalent bonds involving hydrogen atoms were constrained using the SHAKE algorithm,^71^ allowing a 2 fs time step for integrating the equations of motion. Simulations were performed with periodic boundary conditions in the *NPT* ensemble, with *P* = 1 atm and *T* = 310 K, using the Langevin thermostat with a damping constant of 1 ps^–1^ and the Langevin piston^72^ with a constant decay of 50 ps^–1^ and an oscillation period of 50 fs. A flexible unit cell was used with a constant ratio in the *x*–*y* plane. Long-range electrostatic interactions with a cutoff of 12 Å were computed using the particle-mesh Ewald method^73^ with a sixth-order spline and 1 Å grid spacing. To evaluate the models’ performance, five independent 1 μs runs of **M1** and three independent runs for each **M2**-**M5** and **POxPC 2:1** systems were performed, while pure POPC and Pure POPC/8·^1^TLD1433 systems were simulated with one run of 1 μs each (Table S1).

### Experimental Methods

Unless otherwise noted, 1,2-dioleoyl-*sn*-glycero-3-phosphocholine (DOPC), choline chloride, and solvents were obtained commercially and used without further purification. Water for NMR experiments was deionized to ≥18.2 MΩ cm using a Barnstead or Milli-Q system. TLD1433 was synthesized according to a previously published procedure.^74^ ^1^H NMR spectra were recorded on a JEOL 400 MHz spectrometer at the University of Texas at Arlington, with chemical shifts reported in ppm relative to residual solvent peaks.

### ^1^H NMR Analysis of Phospholipids

TLD1433 was irradiated in the presence of lipid under normoxia or hypoxia. Three controls were included: Control I: lipid + light; Control II: TLD1433 + light; and Control III: lipid + TLD1433 in the dark.

Normoxia: Solutions were prepared in 6-dram vials equipped with magnetic stir bars. The test sample and control I and III vials were filled with 10 mL aliquots of freshly prepared 0.2 mg/mL DOPC/choline solution, while control II was filled with 10 mL Milli-Q water. The test sample and control II vials were each charged with 0.5 mg of TLD1433 to afford 50 μM photosensitizer solutions.

Hypoxia: The test sample and controls I–III were prepared as described under normoxic conditions. The vials, fitted with septa, were evacuated and purged with argon 3 times, followed by an additional 30 min argon purge under stirring to ensure deoxygenation.

The control III vial was wrapped in aluminum foil and kept in the dark, while the remaining vials were irradiated with a visible light lamp for approximately 1 h (radiant exposure: 100 J·cm^–2^, irradiance: 27.5 mW·cm^–2^). After irradiation, volatiles were removed, and the test and control samples were further dried *in vacuo*. The resulting solid residues were dissolved in 700 μL of DMSO-*d*_6_ and analyzed by ^1^H NMR spectroscopy.

## Results

### Spontaneous Insertion Mechanism

To expand our understanding on the activity of TLD1433, we focused our investigation on its interaction with lipid membranes at the molecular scale. The amphiphilic nature of TLD1433, arising from the dicationic Ru center combined with the lipophilic oligothiophene chain (Figure 2a), suggests a propensity for membrane association that could influence the structure and dynamics of the lipid bilayer. To explore this behavior, atomistic MD simulations were performed using a single TLD1433 molecule in its ground-state configuration to model dark conditions (**M1**, Figure 1a). Equilibrium MD simulations on the **M1** system show that TLD1433 spontaneously inserts into the membrane within 600 ns in all replicas and remains stably embedded for the duration of the simulations (Figure 2b).

**Figure 2:**
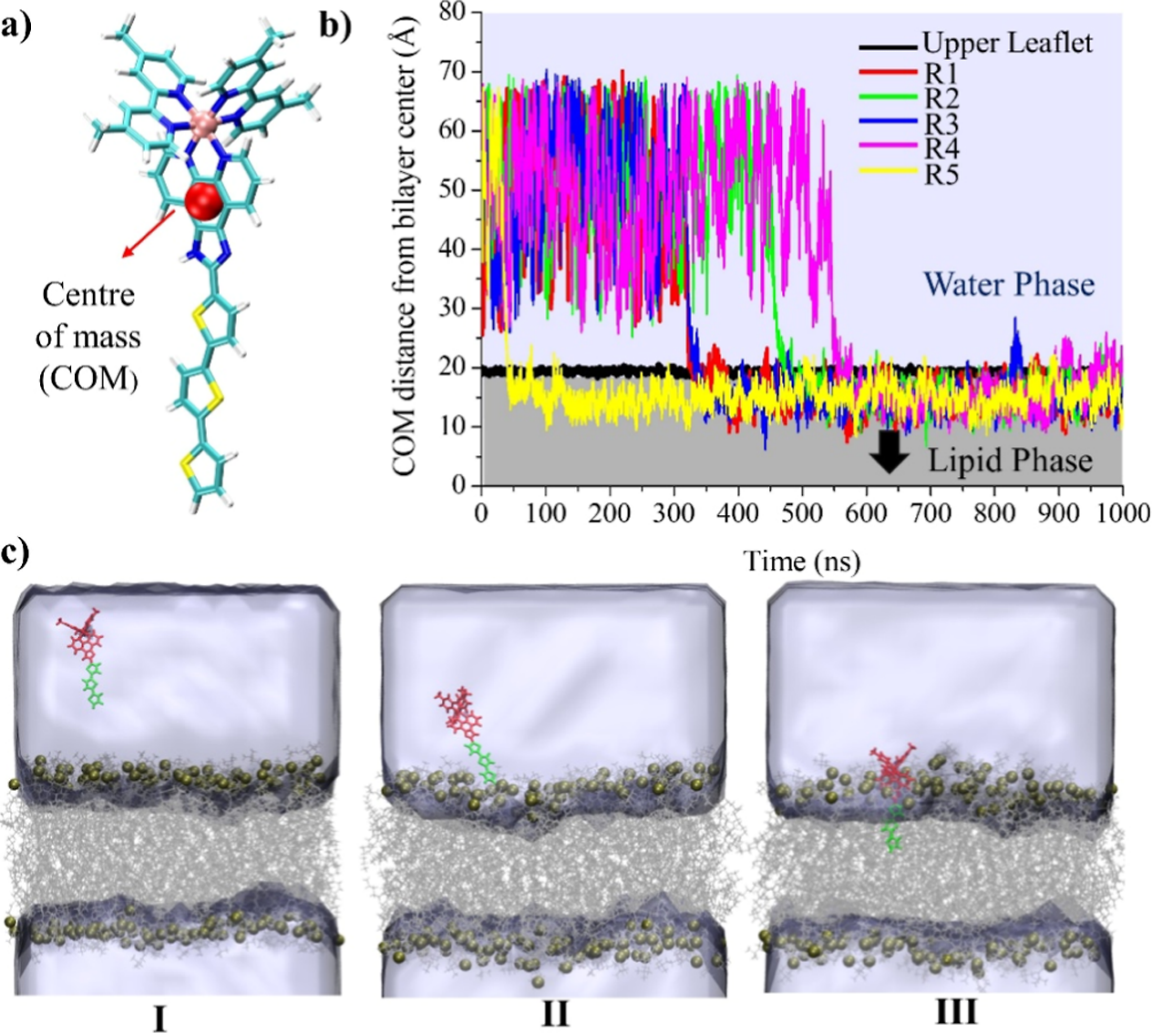
(a) TLD1433 (Ruvidar®) structure and its center of mass (COM); (b) time dependence of the distance between the COM of TLD1433 and the bilayer center in all the simulation replicas R1–R5 (the black line is the center of mass of P atoms of the upper layer of the membrane in one of the trajectories). (c) Snapshots extracted at three different times from MD simulations showing the spontaneous insertion of TLD1433 within the POPC membrane. TLD1433 is shown as licorice with thiophene chain in green, while the rest of the complex in red. POPC lipid phosphorus atoms are shown as tan spheres and acyl chains as gray lines, water is reported as a gray surface.

Three representative snapshots extracted at different simulation times (I–III) are shown in Figure 2c and illustrate the membrane insertion process. At an early stage (Time I), TLD1433 resides in the aqueous phase. As the simulation progresses (Time II), the molecule migrates toward the membrane interface, approaching the bilayer plane primarily through the oligothienyl chain. At Time III, the hydrophobic moiety partitions into the nonpolar region of the membrane, with the oligothienyl chain oriented approximately perpendicular to the bilayer plane and embedded within the membrane core. In contrast, the positively charged metal center remains associated with the negatively charged lipid phosphate headgroups, forming favorable electrostatic interactions (Figure 2c).

The length of the oligothienyl chain is therefore an important structural parameter: increasing the number of thienyl rings enhances lipophilicity, reaching maximal values for *n* = 3 and *n* = 4,^46^ and correspondingly increases the tendency for membrane penetration and localization.

### TLD1433-Membrane Interactions in Normoxia

TLD1433 is a well-established photosensitizer that exhibits strong phototoxic activity under irradiation, with minimal dark toxicity, and has demonstrated efficacy against cancer cells as well as viruses and bacteria.^74,75^ Past studies have suggested that the light-induced lipid peroxidation may contribute to viral inactivation mediated by TLD1433.^76^ In this context, we sought to assess whether oxidized lipid species could be detected in eukaryotic membrane models following treatment with TLD1433 under dark and light conditions.

To this end, ^1^H NMR experiments were performed on a model membrane system consisting of a DOPC/choline mixture (1:1) in the presence of TLD1433 at a concentration of 50 μM (Figure 3a). Upon visible-light irradiation for 1 h under normoxic conditions, new resonances appear in the 11.4–11.2 ppm range, which are consistent with the formation of oxidized lipid species containing carboxylic acid functionalities. Although the present NMR experiments do not permit unambiguous assignment of specific oxidation products, these observations provide clear evidence of lipid oxidation following irradiation in the presence of TLD1433. In contrast, no irradiation-induced byproducts were detected in the corresponding control samples I, II, and III (Figure 3b).

**Figure 3:**
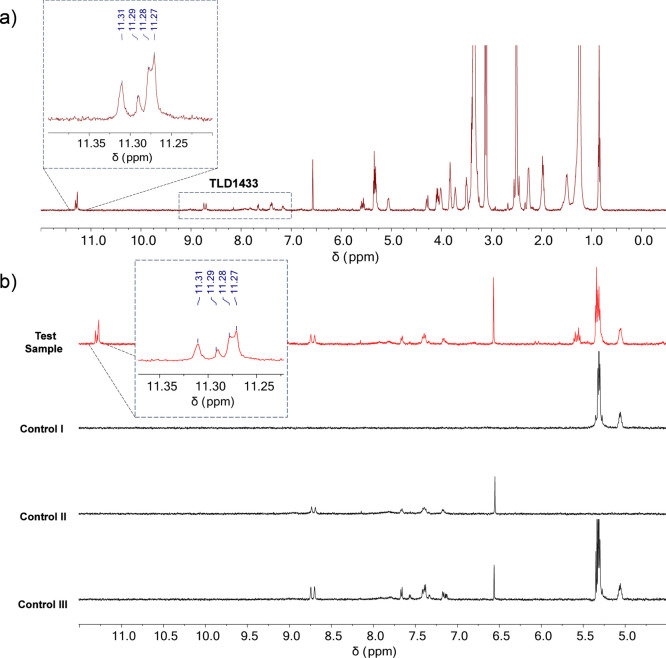
Normoxic conditions: (a) ^1^H NMR spectrum (DMSO-*d*_6_) of a DOPC/choline (1:1 mol/mol) mixture recorded after 1 h of visible-light irradiation (fluence: 100 J/cm^2^; irradiance: 27.5 mW/cm^2^) in an aqueous (Milli-Q) solution (0.2 mg/mL) containing TLD1433 at a concentration of 50 μM. (b) Stacked comparison of ^1^H NMR spectra (DMSO-*d*_6_) of the irradiated sample and three dark controls: (I) DOPC/choline, (II) TLD1433 alone, and (III) DOPC/choline mixture in the presence of TLD1433.

The formation of carboxylic groups is attributed to oxidative attack on the unsaturated lipid acyl chains by singlet oxygen.^77,78^ As polar functionalities, these oxidized lipid species can substantially alter membrane structure and biological function, leading to changes in key biophysical properties of the bilayer.^79^

The impact of membrane alterations induced by TLD1433 under irradiation was quantified using MD simulations by monitoring structural parameters that, when significantly perturbed, have been associated with cellular dysfunction and apoptotic processes.^52,79^ Specifically, these parameters include an increase in the occupied area per lipid, bilayer thinning, and a decrease in lipid chain ordering. POxPC was selected as a representative and well-characterized oxidized phospholipid for the membrane simulations.

To simulate a normoxic environment with a high ROS-mediated lipid oxidation, we used a membrane system with a 2:1 ratio of oxidized to nonoxidized lipids POxPC/POPC as an extreme case of oxidation level. The structural properties of that membrane alone were first characterized in the control model **POxPC 2:1** (Figure 1b and Table S1). The degree of membrane perturbation induced by the light-activated ^3^TLD1433 has been ascertained carrying out simulations in which eight molecules of the drug (four per leaflet) in their triplet excited state were embedded within the same highly oxidized membrane (**M2**) and even considering an inverse POxPC/POPC ratio of 1:2, in model **M3** (Table S1 and Figure 1a). For comparison, a reference system consisting of eight TLD1433 molecules embedded in a pure POPC membrane, with the complex in its ground state (S_0_), was also investigated to simulate dark conditions (**Pure POPC/8·** ^**1**^ **TLD1433**, Table S1 and Figure 1b).

Analysis of the trajectories indicates that aggregation of TLD1433 within the membrane is not observed under the simulated conditions. While it could, in principle, influence membrane association and local membrane perturbation, no evidence for aggregate formation was observed in the molecular dynamics simulations performed here, with the complexes remaining spatially separated throughout the trajectories. Accordingly, the membrane effects reported in this study are attributed primarily to interactions between individual TLD1433 molecules and the lipid bilayer.

The simulations further show that lipid oxidation is associated with the spontaneous emergence of water-permeable pore formation in all replicas of models **M2** and of the control **POxPC2:1** (Figures 4 and S2, respectively). Notably, the presence of TLD1433 in its light-activated triplet state markedly accelerates pore formation, occurring within the first ∼100 ns of the trajectories, whereas similar features emerge only after approximately time-averaged 400 ns in simulations of the oxidized membrane alone (Table S3). As illustrated in Figure 4a, this behavior corresponds to an increase in membrane permeability that facilitates water penetration through transient channels. The red trace, corresponding to the photoactivated complex embedded in the most oxidized membrane, shows both an earlier appearance of pores and a higher degree of water ingress compared to the oxidized membrane alone (black trace).

**Figure 4:**
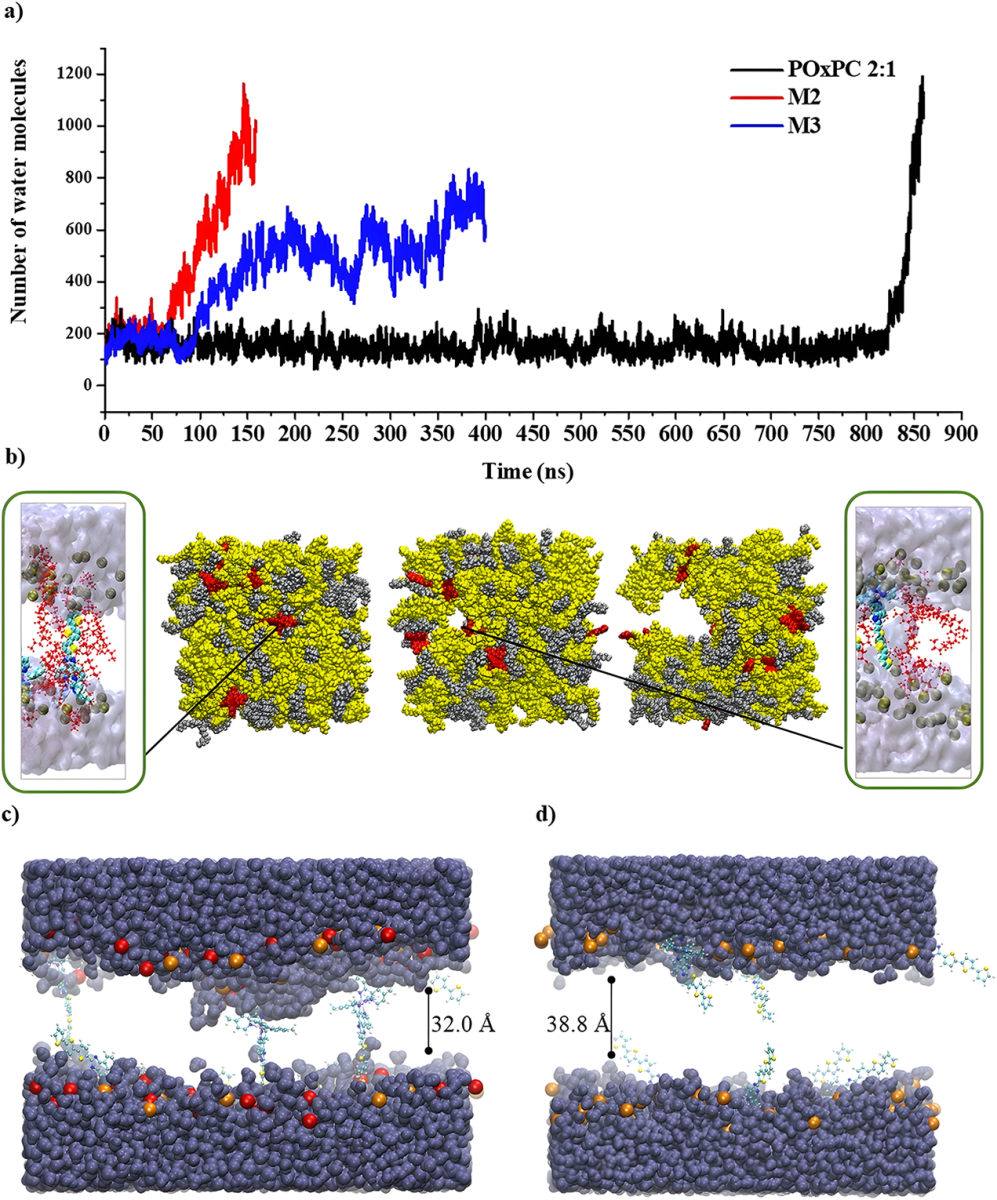
(a) Time evolution of the number of water molecules reaching the center of the lipid bilayer in the simulated systems: **POxPC 2:1** membrane alone (black line), **POxPC 2:1** membrane with 8 embedded TLD1433 in the triplet excited state (**M2**, red line), and **POxPC 1:2** membrane with 8 embedded TLD1433 in the triplet excited state (**M3**, blue line); (b) Representative snapshots extracted from MD simulations illustrating pore evolution in **M2**. TLD1433 is shown in red, POxPC in yellow, and POPC in gray. Water molecules are omitted for clarity. Insets show magnified views highlighting the mechanical action of TLD1433 before (left) and after (right) pore formation. In the insets, POxPC lipids are shown in red; water is depicted as a surface, and POPC lipids are omitted for clarity. (c) Snapshots of MD trajectories showing the structural effects caused by TLD1433 on lipid bilayer upon light excitation (**M2**, **POxPC 2:1/8^.^** ^**3**^ **TLD1433**) and (d) in dark (pure POPC/8^.1^TLD1433). Water molecules are shown as dark blue spheres; phosphorus atoms of POPC are shown in orange, while those of the oxidized lipids are shown in red. The lipid bilayer is omitted for clarity. The vertical black lines indicate the average membrane thickness values. Their placement within the figure is arbitrary.

In Figure 4b, a closer inspection of the pore formation process reveals a higher concentration of oxidized lipids in regions where the membrane defects progressively develop. In these snapshots, such locally accumulated oxidized lipid species are highlighted in red in correspondence with emerging pores. The overall process is accompanied by a decrease in the distance between lipids belonging to opposite leaflets during the simulations, indicative of a local membrane shrinkage (Figure 4c).

The presence of TLD1433 in its light-activated triplet state is associated with a further enhancement of these effects, as the complexes tend to concentrate around the pore edges, accelerating the formation of water channels. It is important to note that the triplet state lifetime of TLD1433 is of several tens of microseconds, far longer than the simulation times employed in this work.^28^ As shown in Figure 4c, photoactivation of TLD1433 produces a local membrane depression and a measurable decrease in bilayer thickness in model **M2** compared to dark conditions. Under irradiation, the mean membrane thickness is reduced to 32.0 Å, whereas a value of 38.8 Å is observed in the dark (Figure 4c,d, respectively). This behavior is consistent with a combined contribution from localized lipid oxidation and TLD1433-membrane interactions that promote membrane deformation. In contrast, in the absence of light, TLD1433 remains associated with the phosphate headgroups while the α-terthienyl chains align with the lipid acyl tails, but no significant membrane thinning or lipid oxidation is observed, in agreement with the low dark cytotoxicity of the compound (Figure 4d).

Pore formation is still observed when the oxidation level is reduced by considering the inverse POxPC/POPC ratio of 1:2 in model **M3**, although it occurs on longer time scales (Figure 4a, blue trace and Figure S3). In this case, all replicas exhibit water channel formation with an average onset time of approximately 200 ns (Table S3), which is slower if compared to the higher oxidized environment but remains faster than in simulations of the same membrane without TLD1433 (**POxPC2:1**). As in **M2**, pore regions are characterized by a local accumulation of light-activated TLD1433 and oxidized lipids (Figure S3). Consistently, the mean membrane thickness under irradiation in **M3** is reduced relative to dark conditions (34.5 Å versus 38.8 Å, respectively).

It should be noted that the two oxidation levels considered in the **M2** and **M3** models (67% and 33%, respectively) should be understood as representing localized oxidation conditions within confined membrane nano regions, rather than bulk-averaged membrane compositions. Instead, they represent regions of highly enriched lipid oxidation arising from spatially clustered ROS activity, as expected under PDT conditions in oxygenated environment.

### TLD1433-Membrane Interactions in Less Oxidized Environments

The formation of membrane pores observed in the MD simulations is associated with light-dependent oxidation of membrane phospholipids mediated by TLD1433. This behavior appears to be strongly oxygen-dependent, as lipid oxidation is required to induce membrane perturbations leading to pore-like defects. Such conditions may be difficult to achieve in hypoxic environments, which are known to limit oxygen availability during photodynamic processes.

Accordingly, alternative or complementary pathways may contribute to the phototoxic activity of TLD1433 under low-oxygen conditions and therefore warrant further investigation. To assess whether lipid oxidation occurs in the absence of oxygen, ^1^H NMR experiments were performed on a DOPC/choline (1:1) mixture in the presence of TLD1433 (50 μM) under hypoxic conditions. As shown in Figures 5 and S4, no resonances attributable to oxidized lipid species are detected in the absence of oxygen, indicating that lipid oxidation is not observed under the hypoxic conditions examined here.

**Figure 5:**
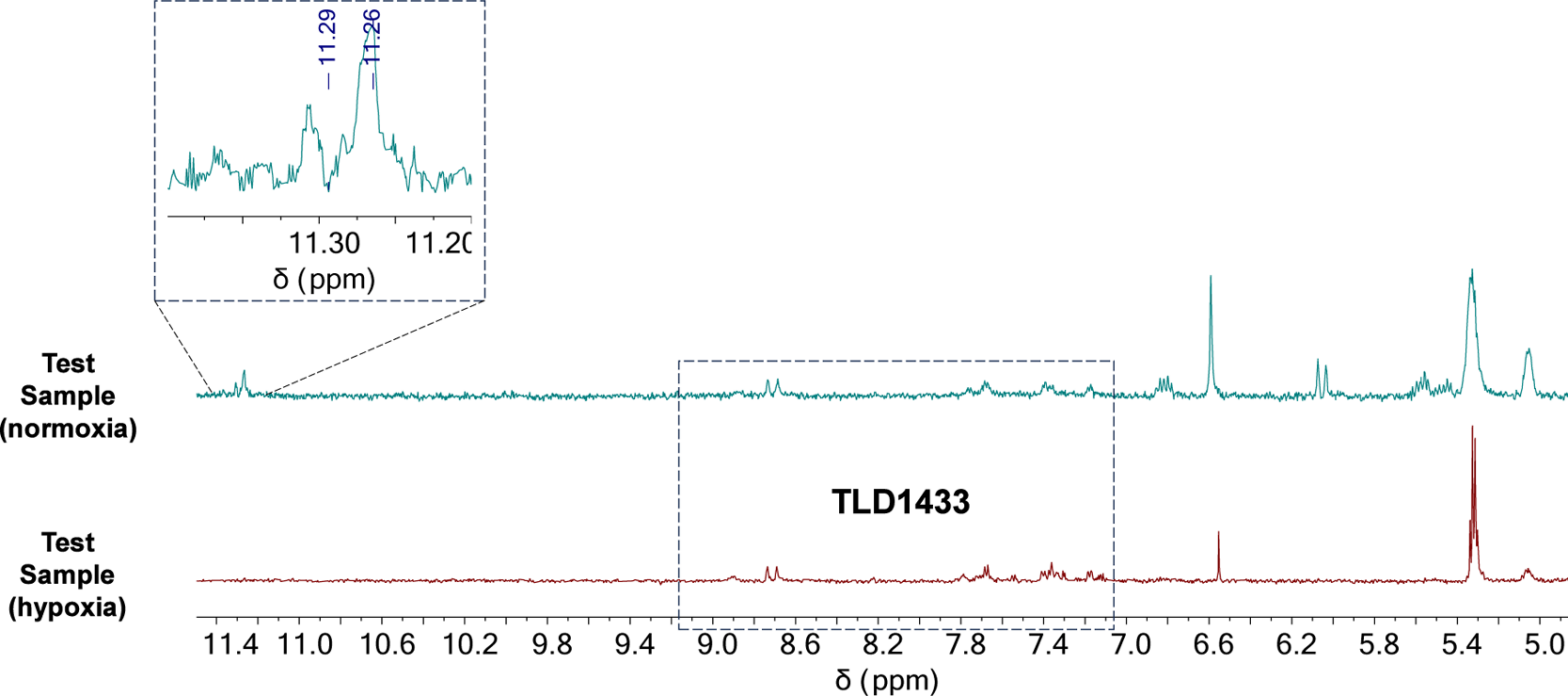
Hypoxic and normoxic conditions. ^1^H NMR spectra (DMSO-*d*_6_) of a DOPC/choline (1:1 mol/mol; 0.2 mg/mL) mixture after 1 h of visible-light irradiation (fluence: 100 J/cm^2^; irradiance = 24.5 mW/cm^2^) in an aqueous solution containing TLD1433 at a concentration of 50 μM. Spectra obtained under normoxic (upper) and hypoxic (lower) conditions are shown for comparison.

To gain insight into the behavior in less oxidized environments, additional MD simulations were carried out using fewer oxidized lipids confined within membrane nanodomains and in proximity to the TLD1433 photosensitizer, thereby modeling progressively lower levels of ROS-mediated lipid peroxidation.

These models are characterized by POxPC/POPC lipid ratios of 1:10 and 1:20 for **M4** and **M5**, respectively (Table S1 and Figure 1a). These systems simulate increasingly severe hypoxic environments under which TLD1433 and several related analogues have been reported to retain phototoxic activity.

Results with both models do not show membrane poration, even when the multiple replicas are simulated up to 1.5 μs. This observation suggests that pore formation is unlikely at very low oxygen tension. In contrast to **M2** and **M3**, the limited number of oxidized lipids in these systems is insufficient to promote the formation of a water channel by facilitating displacement of TLD1433 toward the opposite leaflet (Figure S5). Consistent with this behavior, the ^1^H NMR analysis further corroborates that molecular oxygen is crucial for lipid oxidation.

Nevertheless, membrane poration is not the only process that may contribute to cell death.^53,80–83^ Interaction between drugs and lipid membranes can induce light-driven structural modifications that perturb membrane properties, ultimately leading to cellular dysfunction. Importantly, membrane permeabilization is not necessarily dependent on molecular oxygen. The spontaneous insertion of TLD1433 into the bilayer demonstrates that the complex can penetrate and accumulate within the membrane, giving rise to increased permeability even in the absence of singlet oxygen-mediated lipid oxidation.

To further explore membrane perturbations under both normoxic and hypoxic conditions, membrane structural parameters were analyzed for all systems containing embedded ^3^TLD1433 in oxidized membranes (**M2-M5**) and compared to those obtained in the dark considering pure POPC bilayer (**Pure POPC/8·^1^TLD1433** and **Pure POPC**, Figure 1b and Table S1). Accordingly, the average area per lipid, bilayer thickness, lipid tilt angle, and deuterium order parameter (S_cd_) were calculated, and the results are presented in Figure 6. A comparison of the model systems at different oxidation levels reveals a clear increase in surface area per lipid with increasing lipid oxidation. This trend is illustrated in Figure 6a by Gaussian distributions of different colors, ranging from the light-blue curve corresponding to the pure POPC membrane to the green curve representing the most highly oxidized system (**M2**). The increase in surface area observed for **M2** is associated with the presence of oxidized polar groups, which preferentially orient toward the aqueous phase to interact with water molecules. As oxidation shortens the lipid acyl chains, they adopt a more bent conformation relative to nonoxidized lipids, occupying a larger lateral area within the bilayer. This structural reorganization is reflected in a reduction of the distance between phosphate headgroups of the opposing leaflets and, consequently, in a decrease of membrane thickness with the increase of the oxidation level (Figure 6b, from light-blue to green).

**Figure 6:**
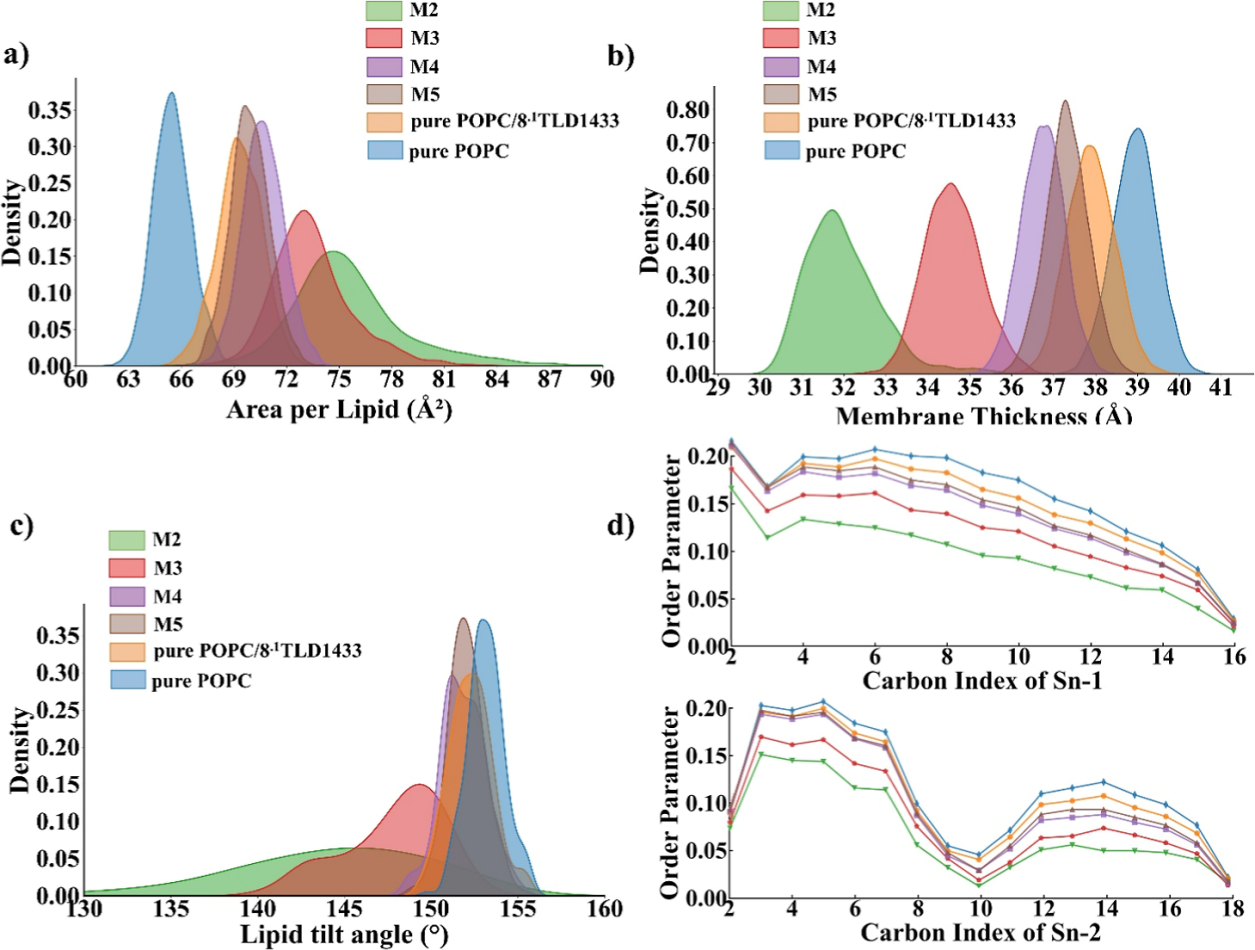
Time dependence of membranes structural parameters for the simulated systems. (a) Area per lipid; (b) average bilayer thickness; (c) lipid tilt angle; (d) deuterium order parameter (S_cd_) for model membranes at different levels of lipid oxidation.

As discussed above, in **M2** and **M3**, the membrane permits sufficient water penetration into the center of the lipid bilayer to create pores. In the remaining models, although stable pores are not observed, the presence of a thinner bilayer can increase water permeability (Figure 6b).

The lipid tilt angle and deuterium order parameter (S_cd_), shown in Figure 6c,d, respectively, exhibit a clear dependence on the POxPC/POPC ratio. The lipid tilt angle reflects the inclination of the acyl chains relative to the healthy bilayer system along the *z*-axis, whereas S_cd_ provides a measure of membrane disorder arising from lipid oxidation induced by embedded compounds.^83,84^ As shown in Figure 6c, increasing oxidation levels occur alongside a progressive increase in lipid tilt angle and a concomitant decrease in the deuterium order parameter, consistent with structural distortion introduced by the polar oxidized groups within the bilayer. This effect is more pronounced for oxidized lipids, owing to their shorter and more flexible chains.

Based on the overall membrane structural analysis, lipid oxidation triggered by the photoreactivity of TLD1433 under normoxic conditions (**M2** and **M3**) is linked to a disruption in membrane organization, manifested by changes in fluidity, morphology, and bilayer thickness, and accompanied by pore formation. These results also complement earlier reports in which TLD1433-mediated photooxidation was associated with damage to lipid membrane-containing viral particles.^76,85^ While those studies demonstrated the biological consequences of lipid oxidation, the present work provides a molecular-level framework linking oxidation-induced membrane remodeling to membrane perturbation and poration. In addition, lipid peroxidation is also recognized as a key biochemical hallmark of ferroptosis, a regulated cell death pathway characterized by the accumulation of oxidized membrane lipids.^87,88^ Given that TLD1433 is capable of Type I photochemistry and generates ROS that promote lipid oxidation, the membrane perturbations observed here may be relevant to ferroptosis-associated mechanisms. However, the present study was designed to investigate membrane-level effects of lipid oxidation and does not directly assess ferroptotic signaling or cell death pathways. Under less oxidized conditions, represented by **M4** and **M5** systems, excitation of TLD1433 induces membrane alterations that do not progress to pore formation but nevertheless result in increased membrane permeability (Figure S6). In these environments, where ^1^H NMR data indicate the absence of detectable lipid peroxidation, the observed increase in membrane permeability suggests that membrane perturbation may nevertheless contribute to the activity of TLD1433 under low-oxygen conditions. Previous studies have demonstrated that TLD1433 and related oligothienyl Ru­(II) complexes retain significant phototoxicity under hypoxic conditions.^28,44^ Accordingly, lipid oxidation-driven membrane poration likely represents one oxygen-dependent component of the overall phototoxic mechanism rather than its sole mode of action, with additional Type I photochemical pathways potentially contributing under oxygen-limited conditions.

## Conclusion

This study integrates atomistic molecular dynamics simulations with experimental evidence of oxygen-dependent lipid oxidation to explore how the photochemical behavior of TLD1433 may influence lipid membrane structure under irradiation. By focusing on molecular-level interactions and membrane responses, our aim was to assess whether light-induced membrane perturbation at the nano scale could plausibly contribute to the phototoxic effects associated with TLD1433-mediated photodynamic therapy.

The simulations indicate that TLD1433 can spontaneously insert and partition into the membrane in the dark, with the hydrophobic α-terthienyl chain aligning with the lipid tails, while the metal center remains associated with the phosphate headgroups. Complementary NMR measurements demonstrate that light irradiation of TLD1433 under normoxic conditions leads to the generation of ROS and oxidation of membrane phospholipids. Consistent with these observations, the computational models show that lipid oxidation promoted by photoactivated TLD1433 is accompanied by a reduction in bilayer thickness and by changes in lipid order and fluidity. At high levels of oxidative perturbation, these effects are associated with increased water penetration and the emergence of pore-like defects in the membrane.

Taken together, these findings suggest that light-induced lipid membrane perturbation, including increased permeability and poration, may represent a contributing factor to the phototoxic activity of TLD1433 under normoxic conditions. Under low levels of oxidation, membrane perturbations remain more limited and do not progress to stable pore formation, although measurable alterations in membrane integrity are still observed and could operate with additional Type I photochemical pathways remaining active under oxygen-limited conditions.

While the present study is based on atomistic simulations and complementary experimental observations rather than direct biophysical measurements of membrane poration in cells, it provides a mechanistically plausible molecular framework that motivates future experimental validation.

Overall, this work provides molecular-level insight into how the photochemical properties of TLD1433 can modulate membrane response in an oxygen-dependent manner, offering a framework for understanding how such effects may contribute to the therapeutic efficacy of TLD1433-mediated PDT and guide the design of next-generation photosensitizers.

## Supplementary Material



## Notes

Model compositions, number of independent replicas and total simulation times for each considered study model and control systems; Snapshots of the evolution of the poration involving **POxPC 2:1** and **1:2** membranes, and time for pore formation; Atomic RESP charges used in the MD simulations of TLD1433 in the S_0_ and T_1_ state; Chemical structures of POPC and POxPC; Representative snapshots of less oxidized **M4** and **M5** models; ^1^H NMR spectrum in hypoxia conditions; Time evolution of the number of water molecules reaching the center of the bilayer in the simulated systems (PDF)
