# Supplementary material for: Oxygen-Dependent Light-Induced Lipid Membrane Perturbation by TLD1433 as a Potential Contributor to Phototoxicity
Source: Inorg Chem. 2026 Jul 20;65(30):17449–61. doi: 10.1021/acs.inorgchem.6c01825 (PMC13439666; doi:10.1021/acs.inorgchem.6c01825)
Supplement: Supplementary file 1 [file ic6c01825_si_001.pdf]

Supporting Information

Oxygen-dependent light-induced lipid membrane perturbation by TLD1433 as a potential contributor to phototoxicity

Alessandra G. Ritacca,[1] Abdelazim M. A. Abdelgawwad,[2] Alisher Talgatov,[3] Colin G. Cameron,[3] Sherri A. McFarland,[3*] Antonio Francés-Monerris,[2*] and Marta E. Alberto[1*]

[1]Dipartimento di Chimica e Tecnologie Chimiche, Università della Calabria, Arcavacata di Rende 87036, Italy.

[2]Institut de Ciència Molecular, Universitat de València, P.O. Box 22085, València 46071, Spain.

[3]Department of Chemistry and Biochemistry, The University of Texas at Arlington, Arlington, Texas, 76019-0065 USA

Table S1. Model compositions, number of independent replicas and total simulation times for each considered study model and control systems…………………………………………………….**S1**

Figure S1. Chemical structures of **POPC** and its oxidative product **POxPC** lipids………………………………………………………………………………………...…**S2**

Table S2: Atomic RESP charges used in the MD simulations of TLD1433 in the S0 and T1 states……………………………………………………………………………………….....**S3**

Figure S2: Representative snapshots of the evolution of the poration involving **POxPC 2:1** membrane……………………………………………………………………………………...**S5**

Figure S3: Snapshot of the pore structure formed in the POxPC/POPC 1:2 membrane (**M3**)…….**S6**

Table S3. Time for pore formation extracted from 3 different MD simulations runs for each POxPC 2:1, M2 and M3 systems……………………………………………………………………**S7**

Figure S4: ^{1}H NMR data…………………………...............……………………….……………..**S8**

Figure S5. Representative snapshots of less oxidised **M4** and **M5** characterized by POxPC/POPC ratios of 1:10 and 1:20, respectively……………………………………………………………. **S9**

Figure S6: Time evolution of the number of water molecules reaching the centre of the bilayer in the M4, M5 and Pure POPC/8^{1}TLD1433 simulated systems, in 1 µs……………………………..**S10**

Table S1. Model compositions, number of independent replicas and total simulation times for each considered study model and control systems. S_0 and T_1 states of TLD1433 are reported as ^{1}TLD1433 and ^{3}TLD1433, respectively.

Study Systems	# POxPC residues	# POPC residues	% Oxidation	# TLD1433 molecules	# MD replicas	Total simulation time (ns)
**M1**<br>Pure POPC/^{1}TLD1433	0	188	0%	1	5	5000
**M2**<br>POxPC 2:1/8·^{3}TLD1433	128	64	67%	8	3	450
**M3**<br>POxPC 1:2/8·^{3}TLD1433	64	128	33%	8	3	1140
**M4**<br>POxPC 1:10/8·^{3}TLD1433	18	180	10%	8	3	4500
**M5**<br>POxPC 1:20/8·^{3}TLD1433	10	200	5%	8	3	4500
**Control systems**						
POxPC 2:1	128	64	67%	0	3	1260
Pure POPC/8·^{1}TLD1433	0	188	0%	8	1	1000
Pure POPC	0	188	0%	0	1	1000

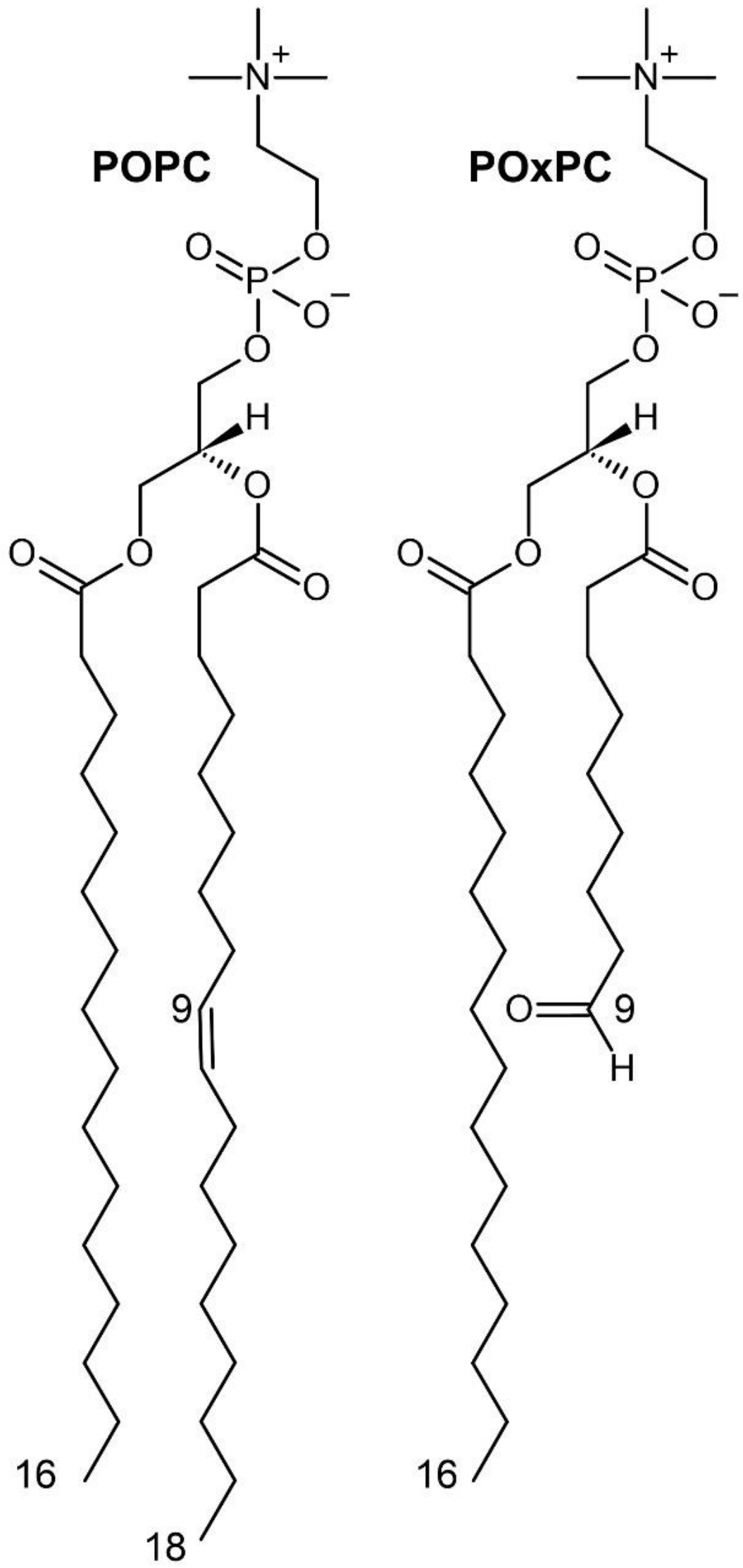

Figure S1. Chemical structures of 1-palmitoyl-2-oleoyl-phosphatidylcholine (**POPC**) and its oxidative product 1-palmitoyl-2-(9′-oxo-nonanoyl)-sn-glycero-3-phosphocholine (**POxPC**) lipids.

Table S2. Atomic RESP charges used in the MD simulations of TLD1433 in the S_0 and T_1 state.

Atom	T_1 state charge	S_0 state charge	Difference
Ru1	-0.8185	-0.7868	0.0317
C1	-0.0502	-0.0587	0.0085
H1	0.1312	0.1358	0.0046
C2	-0.2292	-0.2018	0.0273
H2	0.1749	0.1789	0.0040
C3	-0.0504	-0.0577	0.0073
H3	0.1358	0.1442	0.0085
C4	0.0050	0.0410	0.0359
C5	-0.0136	-0.0596	0.0460
C6	0.3668	0.3580	0.0088
C7	-0.0942	-0.0625	0.0317
C8	0.0027	0.0125	0.0098
H4	0.1342	0.1413	0.0071
C9	-0.2149	-0.2007	0.0142
H5	0.1712	0.1758	0.0046
C10	-0.1037	-0.0766	0.0271
H6	0.1411	0.1371	0.0040
C11	-0.0177	-0.0162	0.0016
C12	0.0023	0.0169	0.0145
C13	0.4930	0.5418	0.0488
C14	-0.0708	-0.1601	0.0893
C15	-0.0554	-0.0268	0.0286
H7	0.1384	0.1377	0.0007
C16	-0.2378	-0.2520	0.0141
H8	0.2148	0.1998	0.0150
C17	0.1547	0.1943	0.0396
C18	-0.1141	-0.1148	0.0006
H9	0.1584	0.1583	0.0002
C19	-0.2164	-0.2098	0.0066
H10	0.1727	0.1749	0.0022
C20	0.3076	0.3072	0.0003
C21	-0.2750	-0.2673	0.0077
H11	0.1683	0.1692	0.0009
C22	0.0907	0.0874	0.0033
C23	0.0907	0.0874	0.0033
C24	-0.2750	-0.2673	0.0077
H12	0.1683	0.1692	0.0009
C25	0.3076	0.3072	0.0003
C26	-0.2164	-0.2098	0.0066
H13	0.1727	0.1749	0.0022
C27	-0.1141	-0.1148	0.0006
H14	0.1584	0.1583	0.0002
C28	-0.1141	-0.1148	0.0006
H15	0.1584	0.1583	0.0002
C29	-0.2164	-0.2098	0.0066
H16	0.1727	0.1749	0.0022
C30	0.3076	0.3072	0.0003
C31	-0.2750	-0.2673	0.0077
H17	0.1683	0.1692	0.0009
C32	0.0907	0.0874	0.0033
C33	0.0907	0.0874	0.0033
C34	-0.2750	-0.2673	0.0077
H18	0.1683	0.1692	0.0009
C35	0.3076	0.3072	0.0003
C36	-0.2164	-0.2098	0.0066
H19	0.1727	0.1749	0.0022
C37	-0.1141	-0.1148	0.0006
H20	0.1584	0.1583	0.0002

N1	0.1536	0.1479	0.0056
N2	0.2212	0.1997	0.0215
N3	-0.4437	-0.4076	0.0361
N4	-0.5760	-0.5946	0.0186
N5	0.1430	0.1413	0.0017
N6	0.1430	0.1413	0.0017
N7	0.1430	0.1413	0.0017
N8	0.1430	0.1413	0.0017
S1	-0.1571	-0.1390	0.0181
H21	0.3994	0.3872	0.0122
C38	0.0705	-0.0167	0.0872
C39	-0.1817	-0.1419	0.0398
S2	-0.1103	-0.0798	0.0304
C40	-0.1497	-0.1909	0.0411
H22	0.1972	0.1800	0.0172
C41	0.1572	0.1327	0.0245
H23	0.1733	0.1662	0.0071
C42	-0.0690	-0.0765	0.0075
C43	-0.0051	-0.0005	0.0046
S3	0.0296	0.0155	0.0141
C44	-0.2665	-0.2806	0.0141
H24	0.1627	0.1416	0.0210
C45	-0.0526	-0.0982	0.0456
H25	0.1947	0.1882	0.0066
H26	0.1952	0.1931	0.0021
C46	-0.4344	-0.4377	0.0032
H27	0.1454	0.1486	0.0033
H28	0.1454	0.1486	0.0033
H29	0.1454	0.1486	0.0033
C47	-0.4344	-0.4377	0.0032
H30	0.1454	0.1486	0.0033
H31	0.1454	0.1486	0.0033
H32	0.1454	0.1486	0.0033
C48	-0.4344	-0.4377	0.0032
H33	0.1454	0.1486	0.0033
H34	0.1454	0.1486	0.0033
H35	0.1454	0.1486	0.0033
C49	-0.4344	-0.4377	0.0032
H36	0.1454	0.1486	0.0033
H37	0.1454	0.1486	0.0033
H38	0.1454	0.1486	0.0033

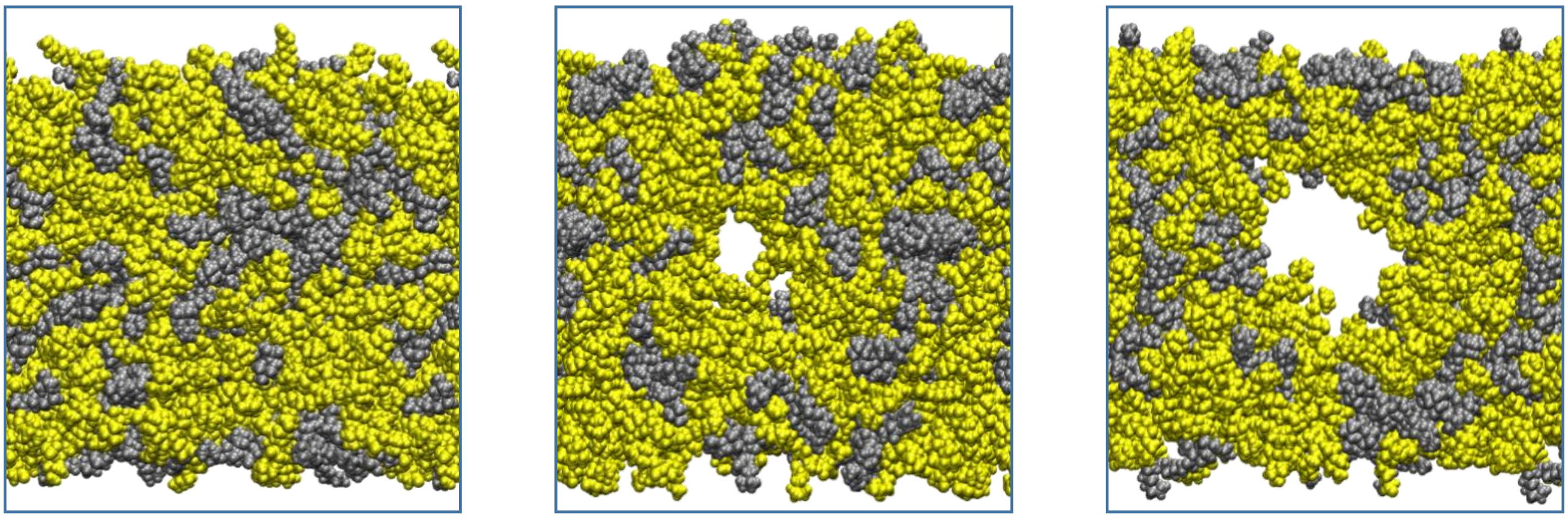

Figure S2: Representative snapshots of the evolution of the poration involving **POxPC 2:1** membrane. POxPC in yellow and POPC in grey. Water molecules are not shown for clarity.

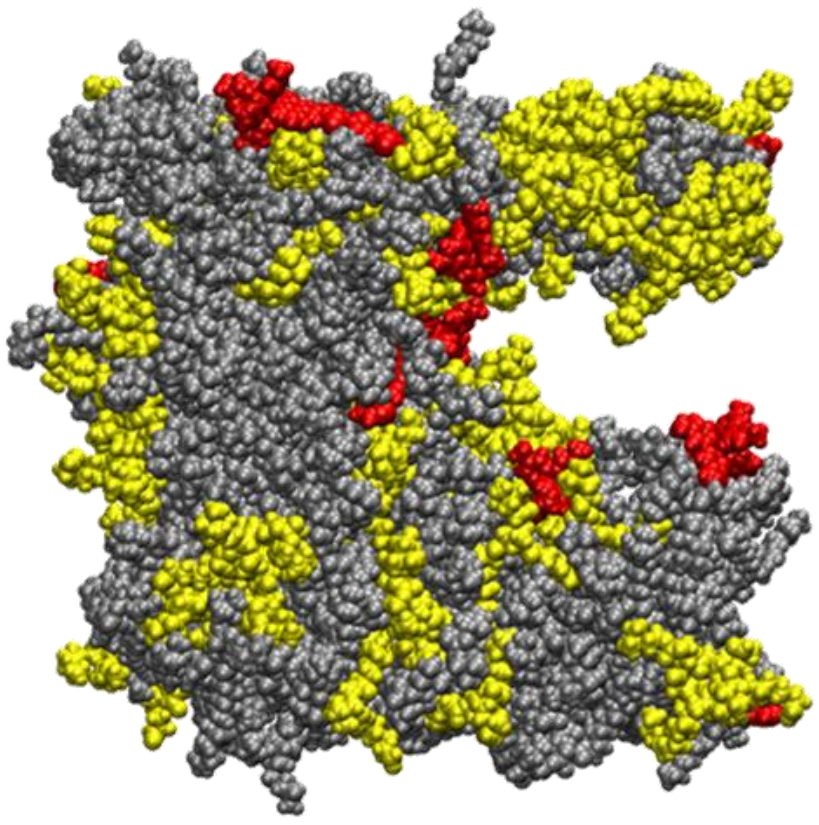

Figure S3: Snapshot of the pore structure formed in the POxPC/POPC 1:2 membrane (**M3**). POxPC in yellow and POPC in grey. TLD1433 are reported in red. Water molecules are not shown for clarity. The image shows the local accumulation of oxidized lipids in correspondence to the pore.

Table S3. Time for pore formation extracted from 3 different MD simulations runs for each POxPC 2:1, M2 and M3 systems.

Systems	POxPC/POPC ratio	n. lipids POxPC/POPC	Time of pore formation
**POxPC 2:1**<br>Run1<br>Run2<br>Run3	**2:1**	128:64	≈830 ns<br>≈140 ns<br>≈200 ns
**M2**<br>Run 1<br>Run 2<br>Run 3	**2:1**	128:64	≈80 ns<br>≈60 ns<br>≈30 ns
**M3**<br>Run 1<br>Run 2<br>Run 3	**1:2**	64:128	≈330 ns<br>≈140 ns<br>≈100 ns

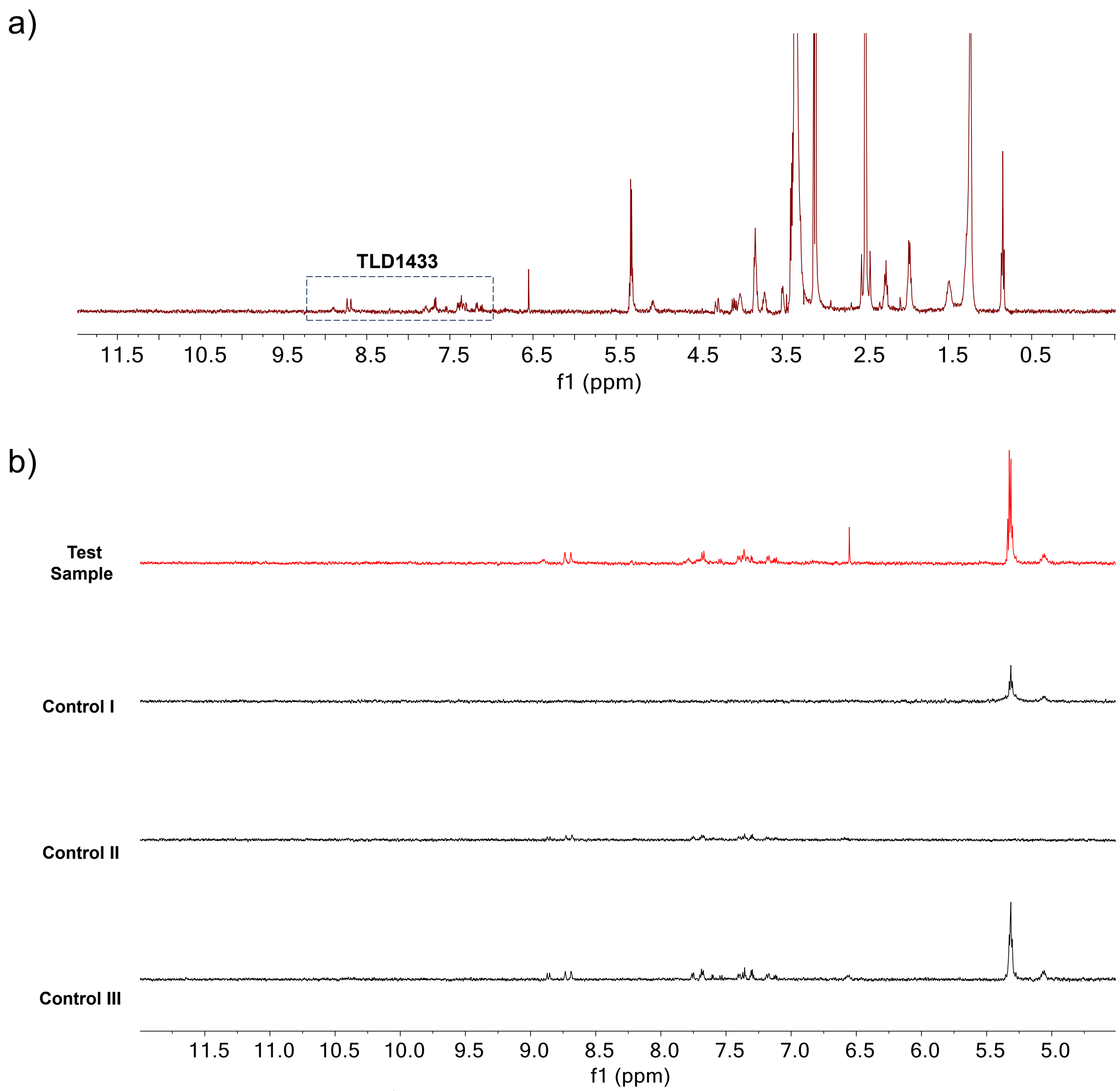

Figure S4: *Hypoxia conditions*. a) ^{1}H NMR spectrum (DMSO-d_6) of a DOPC/choline 1:1 (mol:mol) mixture after 1 hour 8 min 2 sec irradiation at visible light (fluence – 100 J/cm^2, irradiance – 24.5 mW/cm^2) as a 0.2 mg/mL aqueous (Milli-Q) solution in the presence of a 50 μM concentration of TLD1433. In b) b) ^{1}H NMR overlay (DMSO-d_6) of a sample after the irradiation over three controls (I, II and III as DOPC/choline, TLD1433 alone and their mixture, respectively) in dark conditions. No irradiation byproducts are formed after irradiation.

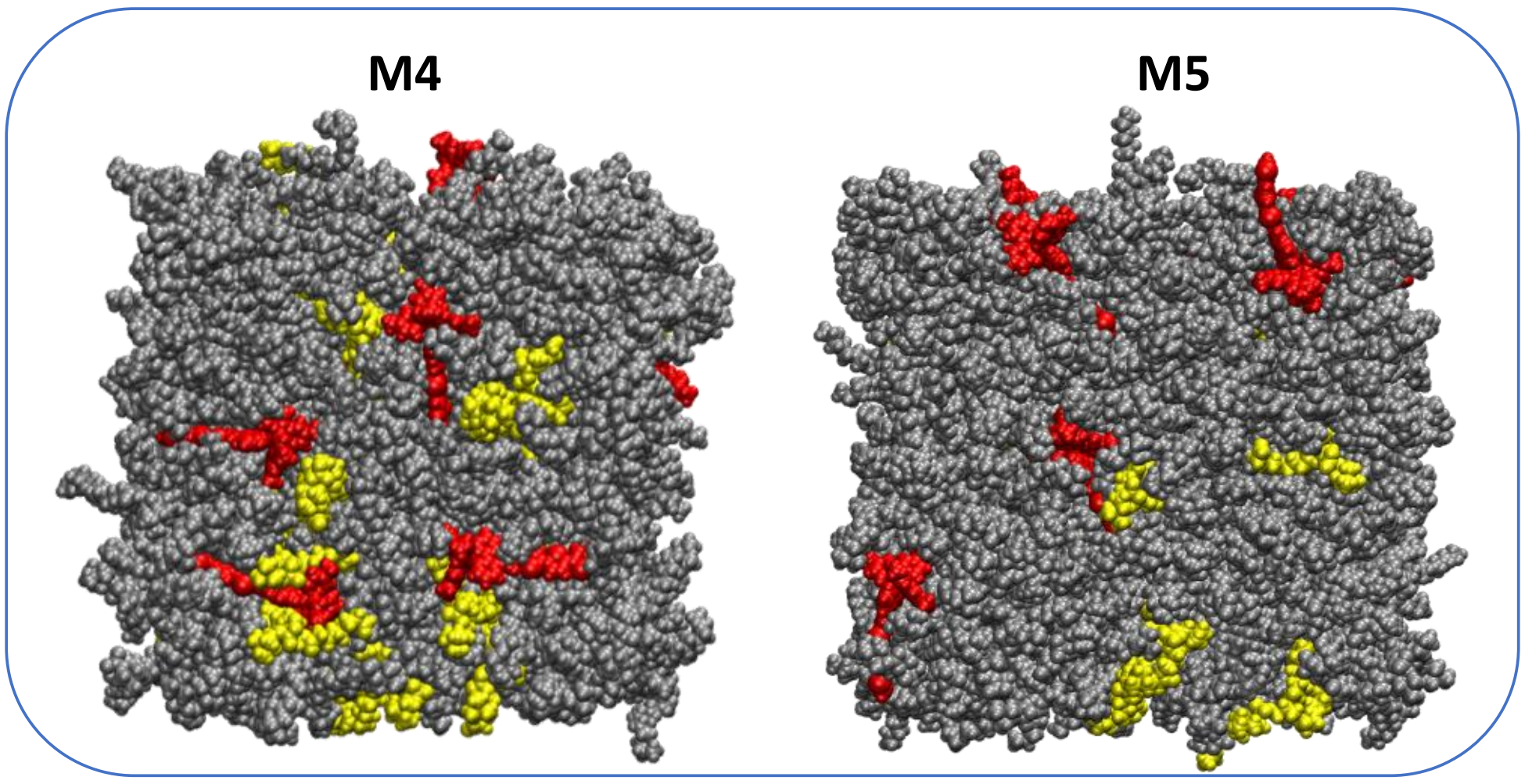

Figure S5: Representative snapshots of less oxidised **M4** and **M5** characterized by POxPC/POPC ratios of 1:10 and 1:20, respectively. The 8 molecules of TLD1433 are reported in yellow, while POxPC are in red; POPC lipids are the grey ones.

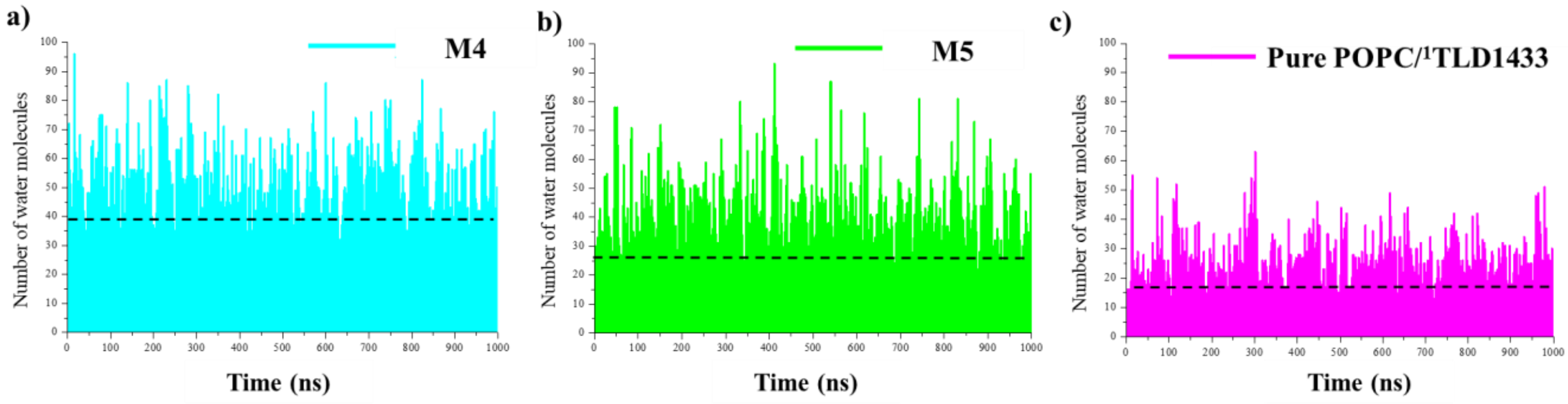

Figure S6: Time evolution of the number of water molecules reaching the centre of the bilayer in the simulated systems, in 1 µs : a) **M4** (POxPC1:10/^{3}TLD1433), b) **M5**: (POxPC1:20/^{3}TLD1433), c) **Pure POPC/8^{1}TLD1433**. Centre of the bilayer: region within the lipid bilayer delimited by the two xy planes defined at z - 10 and z + 10 A, respectively, in which z corresponds to the z component of the lipid bilayer center of mass, which is fixed throughout all simulation.
